# Isolation, Characterization, and RP-HPLC Estimation of P-Coumaric Acid from Methanolic Extract of Durva Grass (*Cynodon dactylon* Linn.) (Pers.)

**DOI:** 10.1155/2015/201386

**Published:** 2015-02-18

**Authors:** Ramadoss Karthikeyan, Chapala Devadasu, Puttagunta Srinivasa Babu

**Affiliations:** Vignan Pharmacy College, Vadlamudi, Guntur, Andhra Pradesh 522 213, India

## Abstract

P-coumaric acid is a nonflavonoid phenolic acid and is a major constituent of the species *Cynodon dactylon* Linn. (Pers.). In this study isolation of P-coumaric acid was achieved by preparative TLC and the compound thus isolated was characterised by UV, mass, and H^1^ NMR spectral analysis. An isocratic RP-HPLC method was developed for the estimation of P-coumaric acid from methanolic extracts of durva grass. The chromatographic separations were achieved by RP-C_18_ column (250 mm × 4.6 mm, 5 *μ*), Shimadzu LC-20AT Prominence liquid chromatograph, and a mobile phase composed of water : methanol : glacial acetic acid (65 : 34 : 1 v/v). The flow rate was 1.0 mL/min and the analyses of column effluents were performed using UV-visible detector at 310 nm. Retention time of P-coumaric acid was found to be 6.617 min. This method has obeyed linearity over the concentration range of 2–10 *μ*g/mL and the regression coefficient obtained from linearity plot for P-coumaric acid was found to be 0.999. RP-HPLC method was validated in pursuance of ICH guidelines.

## 1. Introduction

It has been estimated that there are approximately 8,000 naturally occurring phenolic compounds. Phenolic antioxidants are believed to provide a protective effect against oxidative damage diseases such as cancer, coronary heart disease, and stroke [1–4] and it has antidiabetic action [5, 6]. P-coumaric acid is a hydroxy cinnamic acid, an organic compound that is a hydroxy derivative of cinnamic acid [7, 8]. P-coumaric acid can be found in* Gnetum cleistostachyum* [9]. In food P-coumaric acid can be found in a wide variety of edible plants such as peanuts, navy beans, tomatoes, carrots, and garlic. It is found in wine and vinegar [10]. It is also found in barley grain [11]. P-coumaric acid has antioxidant properties and is believed to reduce the risk of stomach cancer [12], by reducing the formation of carcinogenic nitrosamines [13].

They have excellent antioxidant activities, which are higher than those of vitamins C and E against reactive oxygen species [14]. They have a wide range of biological activities, such as protection against coronary heart diseases, anti-inflammatory, antitumour, antimutagenic, and antimicrobial activities [15].

A thorough literature survey was done to study analytical methods developed so far; it reveals that there were few HPTLC densitometry methods for the determination of P-coumaric acid with other phenolic acids in flowers and roots of the plant sources [16–18], few HPLC-UV detection methods using different mobile phase combinations for the pharmacokinetic study of P-coumaric acid in mouse after oral administration and determination of P-coumaric acid along with other phenolic acids present in various herbs [19–24]. There were few miscellaneous methods reported for determination of phenolic acid content [25–27].

Though several methods have been developed to determine P-coumaric acid in various sample matrices they are probably with high run time, utilise amine additives, and require tedious extraction and sample preparation for analysis in biological fluids and other sample matrices. This emolliates the author to develop an accurate and specific reversed phase high performance liquid chromatography method for determination of P-coumaric acid content in methanolic extract of durva grass with good linearity, and less solvent consumption resulted from the less run time set in the method. This method has been validated according to ICH guidelines [28]. This study was aimed at development of suitable extraction and isolation procedure which aids in getting the purified material and in developing an isocratic RP-HPLC method for estimation of P-coumaric acid in methanolic extract of durva grass. Further the study also deals with characterisation of P-coumaric acid in the extract by mass and H^1^ NMR spectral analysis for structure identification.

## 2. Materials and Methods

### 2.1. Apparatus

A continuous Soxhlet apparatus (18 cm × 4.8 cm, Rankem, Mumbai) with heating mantle (1000 mL capacity) and vacuum filtration assembly (Baba, Mumbai) is used for extraction purpose.

### 2.2. Instruments

Chromatographic separations were achieved by using Shimadzu LC-20AT Prominence Liquid chromatograph comprising a LC-20AT VP pumps, Shimadzu SPD-20A Prominence UV-Vis detector, and Welchrom C18 column (4.6 mm i.d. × 250 mm, 5-micron particle size). 20 *μ*L of sample was introduced into the HPLC system. The HPLC system data acquisition was performed with “Spinchrome” software. Double beam UV-visible spectrophotometer (Systronics model 2203) with matched cuvettes was used in this study. In addition, digital pH meter (Systronics model 802) is used for all pH measurements in this study. An electronic balance (ESSAY TX223L) and an ultrasonic bath sonicator (spectra lab, UCB 40) are also used in the present work.

### 2.3. Chemicals and Reagents

**Scheme 1:**
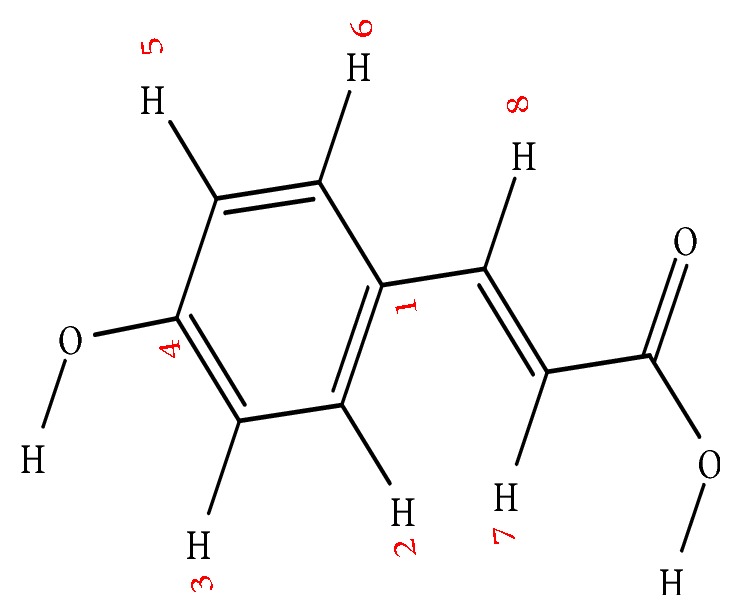
Structure of P-coumaric acid.

The P-coumaric acid was procured from Sigma Aldrich (Scheme 1). Methanol, anhydrous sodium sulphate, calcium chloride, and so forth, were purchased from Merck Pvt. Ltd., Mumbai, India. All the other chemicals used including the solvents were of analytical grade. Glacial acetic acid, Methanol and water (HPLC grade) are also purchased from (Sigma Aldrich, Mumbai).

### 2.4. Collection and Identification of Plant Materials

The plant specimen was collected from medicinal garden of Vignan Pharmacy College, Vadlamudi village, Guntur district, Andhra Pradesh, India. The plant material was collected in the month of July 2014. The specimen was identified and authenticated by Dr. S. M. Khasim, Associate Professor at the Department of Botany and Microbiology, Acharya Nagarjuna University, Nagarjuna Nagar, Guntur, Andhra Pradesh, India. The whole plant was used for the research work.

### 2.5. Preparation of Reagents and Standards

#### 2.5.1. Mobile Phase

65 parts of water, 34 parts of methanol, and 1 part of glacial acetic acid were mixed to get one litre of the mobile phase. The mobile phase was then filtered through 0.22 *μ*m nylon membrane vacuum filtration and degassed by sonication.

#### 2.5.2. Preparation of Standard Solutions

A standard stock solution was prepared by dissolving 100 mg of P-coumaric acid in 100 mL volumetric flask containing 60 mL mobile phase and then sonicated for about 10 minutes and made up to 100 mL with mobile phase to get the primary standard stock solution containing 1000 *μ*g/mL of P-coumaric acid. Working standard solutions were prepared by further dilution with mobile phase.

#### 2.5.3. Preparation of Plant Extract

The whole plant* Cynodon dactylon* was used in this study. The material is cleaned and set free from moulds, insects, animal faecal matter and other contaminations such as earth, stones, and extraneous materials. The specimen was shade-dried and protected from sun light for several days not less than one month. It was ground to a fine powder using mortar and pestle without any loss of powdered drug. Then it was passed through a sieve of 40 meshes and the material passed by the sieve was collected and stored in a well tight amber coloured container and it was used for further study.

Coarsely powdered aerial parts of the plant (about 1 Kg) are successively extracted with continuous Soxhlet apparatus with methanol for 48 hours. The homogenate was filtered using Whatman's filter paper and the volume of the filtrate was recorded. The filtrate was centrifuged at 100 ×g for 15 min under cooling (4–6°C) conditions. The clear supernatant was taken on rotary evaporator and the extracts were concentrated, dried, and stored in vacuum desiccators. Further the extracts were used for different studies.

### 2.6. Preliminary Phytochemical Screening

The main objective of the preliminary phytochemical screening is to investigate the plant extract in terms of its active constituents. It involves the partial isolation of active constituents and identifies them qualitatively. In this screening various types of identification tests for a variety of chemical classes have been performed according to CCRAS guidelines [29].

### 2.7. Isolation and Purification of Active Compounds

#### 2.7.1. Analytical TLC

Analytical TLC was carried out on preparative TLC plates (5 × 5 cm with 0.2 mm thickness, silica gel GF_254_, Merck, Darmstadt, Germany) cut from the commercially available sheets. An aliquot of standard solution of P-coumaric acid and a sample solution of crude extract are spotted onto the silica gel plate and allowed to dry for a few minutes. Afterwards, the chromatoplate was developed with chloroform : methanol : formic acid (85 : 10 : 5 v/v) as mobile phase in a previously saturated glass chamber with eluting solvents for some time at room temperature. The developed plate was dried under normal air and the spots were visualized by spraying with a solution of 0.5% w/v ferric chloride and dried under oven. The Rf (retention factor) values of isolated compounds and standard were calculated and compared.

#### 2.7.2. Preparative TLC for Purification

A streak of crude extract was applied manually on a preparative TLC glass plate (20 cm × 20 cm; 1500 *μ*m thickness) with inorganic fluorescent indicator binder (Analtech, Sigma-Aldrich, Steinheim, Germany). After air drying, the plate was developed, using the same mobile phase as used in the analytical TLC, in a presaturated glass chamber. In each experiment, two plates were used in parallel. One of the plates from each set of experiment was sprayed as described above, and the bands were scraped off carefully from the plate. The scratched sample was dissolved in HPLC grade methanol and centrifuged at 12000 rpm for 15 min in order to remove silica. The supernatant was collected, filtered from 0.22 *μ*m filter, and dried under reduced pressure. Further, all the dried samples were passed under nitrogen gas for 5 min and then dissolved in methanol for further characterization and quantitative HPLC analysis. The entire purification process was carried out under dark or dim light conditions.

### 2.8. Characterization of Purified Compound

The UV spectrum of the purified compound was recorded from 190 to 600 nm on an* ELICO* double beam UV-visible spectrophotometer. ESI mass spectra were acquired from isolated compound and characterised. Proton nuclear magnetic resonance spectra were acquired using NMR spectrometer (400 MH_Z_) employing TMS as an internal standard and deuterated methanol was used as solvent.

### 2.9. Determination of P-Coumaric Acid in Methanolic Extract of* Cynodon dactylon* by RP-HPLC

The chromatograph was stabilised for about 45 minutes with mobile phase consisting of water : methanol : glacial acetic acid (65 : 34 : 1 v/v). The flow rate was 1.0 mL/min phase at the required flow rate to get a steady base line. Aliquots of standard solution containing P-coumaric acid (0.2–1.0 mL, 100 *μ*g/mL) were transferred to a series of 10 mL capacity volumetric flasks to get the concentrations ranging 2–10 *μ*g/mL. Accurately about 20 *μ*L of each calibration standard was injected into the chromatogram. Peak areas of each solution were recorded. A calibration curve was plotted between concentration and peak area response. 20 *μ*L of sample solution prepared from methanolic extract was injected and the area of peak was recorded duly maintaining the ambient experimental conditions as followed by the standard drug solutions. The amount of P-coumaric acid present in the sample of extract was computed from its calibration graph.

#### 2.9.1. Validation of the Developed Method

*System Suitability*. The chromatograph was stabilised for about 45 minutes with mobile phase at the required flow rate to get a steady base line. System suitability was ascertained by six replicate analyses of the drugs at concentrations of 10 *μ*g/mL of P-coumaric acid. The percentage of RSD of the three injections of the same quantity of standard drug solutions of P-coumaric acid in terms of their peak areas, retention time, efficiency, or number of theoretical plates and asymmetry factor ascertains its system suitability for compatibility of analysis and for obtaining reproducible value. The effect of wide range of other constituents and other additives usually present in the extract was investigated to know the specificity of the method. It shows no interference from other compounds. For linearity, aliquots of primary working standard solutions containing P-coumaric acid were diluted in a way such that the final concentrations of P-coumaric acid are in the range of 2–10 *μ*g/mL. A calibration curve was plotted between concentration and peak area response and statistical analysis of the calibration curve was performed. Method of least square analysis was carried out for getting the slope, intercept and correlation coefficient, and regression data values. Precision was determined by intraday and interday study. Precision of the method was evaluated by carrying out the assay and analyzing corresponding responses 6 times on the same day and on different days for the sample solution. Accuracy studies were performed for P-coumaric acid at three different levels (25%, 50%, and 100%) and the mixtures were analyzed in triplicate by the proposed method. A known amount of standard P-coumaric acid at 25%, 50%, and 100% of sample (which was previously analysed) was added and it was reanalysed by the proposed method. And the percentage recovery was evaluated. The robustness of the developed method was evaluated by small deliberate changes in flow rate (±0.1 mL/min), detection wavelength (±5 nm), and mobile phase composition (±2%). The effect of these variables on the developed method was determined. Limit of detection and limit of quantification were calculated using the following formula LOD = 3.3 (*σ*)/*S* and LOQ = 10 (*σ*)/*S*, where (*σ*) = standard deviation of response (peak area) and *S* = slope of the calibration curve.

## 3. Results and Discussion

**Figure 1:**
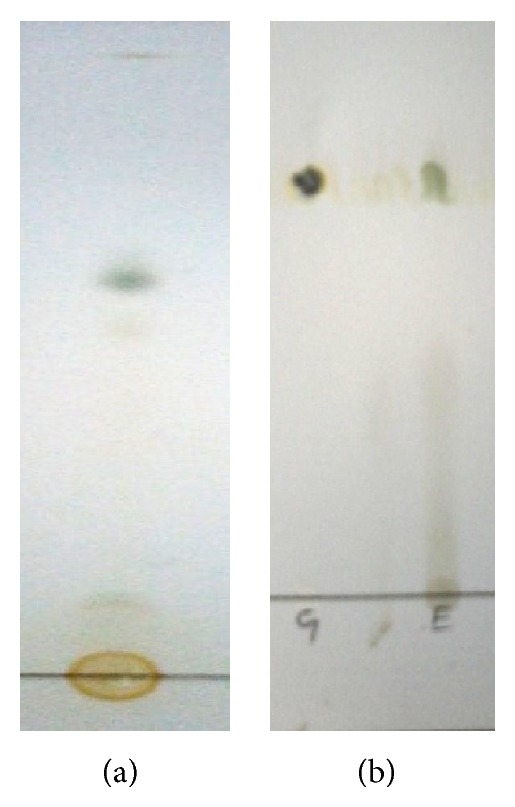
Identification of P-coumaric acid on TLC plate: (a) chromatogram showing identification of P-coumaric acid standard; (b) separation of P-coumaric acid in methanolic extract of durva grass.

**Figure 2:**
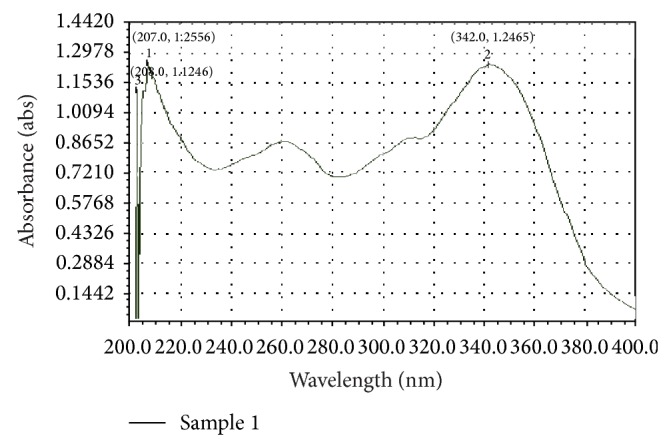
UV absorption spectrum of P-coumaric acid in methanol (isolated from methanolic extract by preparative TLC).

The extraction procedure was optimized regarding extraction solvent and recovery. Methanol was used for extraction of the P-coumaric acid from* Cynodon dactylon* L. Complete extraction of P-coumaric acid was achieved by successive solvent extraction with methanol. Methanol has a protective role. It can prevent phenolic compounds from being oxidized by enzymes, such as phenoloxidases [30, 31]. Preliminary phytochemical study reveals that the extract may contain phenolic compounds which may be non-flavonoid in nature. Several mobile phase combinations were tried and chloroform : methanol : formic acid (85 : 10 : 5 v/v) was found optimum for separation of P-coumaric acid from methanolic extract of durva grass. The RF values of standard and sample compound match each other and the RF value was found as 0.52. This compound is structurally related to the investigated compounds and behaves similarly on the column as analyte. TLC profile of compound was represented in Figure 1. Isolation of P-coumaric acid from the extract was achieved by preparative thin layer chromatography using the same chromatographic conditions followed by identification of active constituent. Characterisation of isolated compound was done by studying ultraviolet, mass, and H^1^ NMR spectra. P-coumaric acid shows UV absorption at about 345 nm in methanol indicates the presence of conjugation and hydroxyl auxochrome which shifts the absorption RF maximum towards visible side of the spectrum and it was represented in Figure 2. Mass spectral data shows molecular ion peak *m*/*z* = 164.2 which has a moderate abundance (Table 1). Spectrum obtained from the H^1^ NMR shows different kinds of protons and its assignment corresponds to type of hydrogen in the complete structure of P-coumaric acid and it was reported in Table 2.

**Figure 3:**
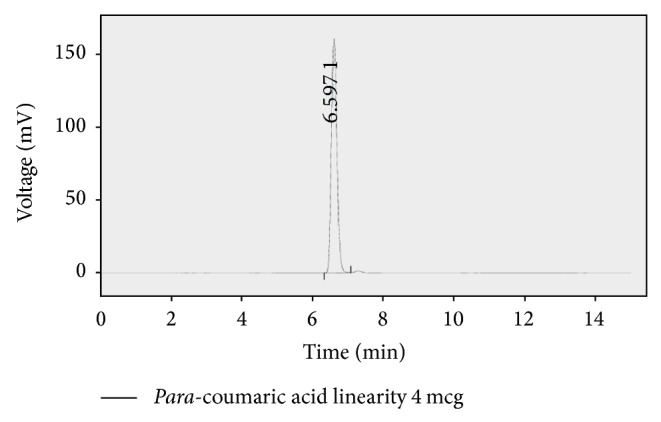
Chromatogram of P-coumaric standard.

**Figure 4:**
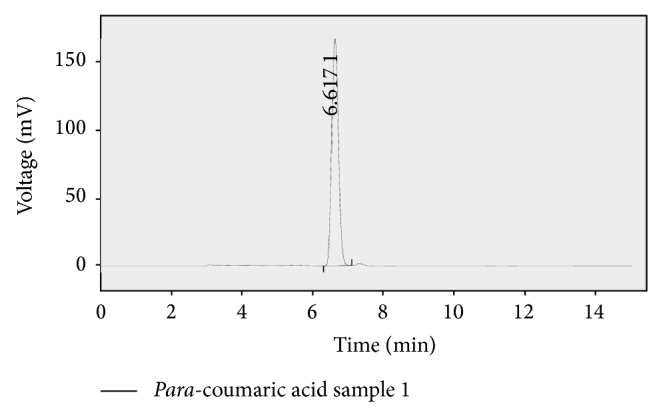
Chromatogram of methanolic extract of P-coumaric acid in durva grass.

**Figure 5:**
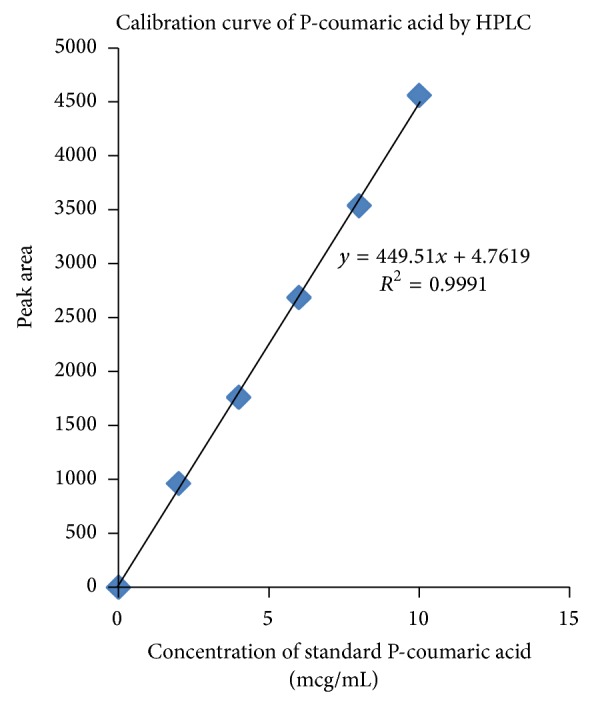
Linearity plot for P-coumaric acid.

An accurate isocratic RP-HPLC method was developed and validated by optimised chromatographic conditions. The conditions and system suitability were presented in Table 3. Chromatograms showed a peak of P-coumaric acid at retention time of 6.617 min. The regression coefficient obtained from linearity plot for P-coumaric acid was found as 0.999, which indicates this method had good linearity and the linearity data was given in Table 4. The representative chromatograms of this method were given in Figures 3 and 4, for calibration standard and sample of methanolic extract, respectively. The calibration plot for P-coumaric acid was shown in Figure 5. The amount of P-coumaric acid was found as 0.48 mg/100 mL extract. The method validation parameters were established in this work, LOD and LOQ of the P-coumaric acid were found as 0.302 *μ*g/mL and 0.99 *μ*g/mL, and the proposed method was found to be precise for the determination. The percentage of RSD for the proposed method was found to be less than 2.0 which indicate the method's precision. Results of the precision study are shown in Table 5. Recovery studies of the method were found to be good and percentage of recovery was represented in Table 3. Robustness was done by small changes in the chromatographic conditions like mobile phase flow rate, *λ*_max⁡_, and mobile phase composition. The proposed method was found to be robust as there were no marked changes in the performance characteristics of the method.

## 4. Conclusion

Isolation, identification, and characterisation of P-coumaric acid was achieved successfully which will be helpful for the standardization of herbal formulations containing this active constituent. The proposed HPLC method is linear, sensitive, accurate, and precise and can be adopted for the determination of concentration of P-coumaric acid in various samples from various herbs and formulations with shorter run time and good efficiency.

## Figures and Tables

**Table 1:** Molecular weights (MW) and important ions present in the mass spectra of P-coumaric acid in the examined plant extracts by ESI-MS.

Compound	Molecular weight (MW)	Molecular ion [M]^+^	Fragmented ions (*m*/*z*)
P-coumaric acid	164.2	164.2	242 (100), 178 (43), 120 (12.2), 40 (19)

**Table 2:** Chemical shifts of P-coumaric acid in H^1^ NMR spectra, *δ* (ppm).

Proton number	Chemical shift (*δ*_ppm_)	
	Theoretical [29, 30]	Experimental (isolated compound)
1	12.13	—
2	8.10	7.341
3	6.97	6.689
4	3.56	3.219
5	7.06	6.725
6	7.65	7.364
7	6.44	6.199
8	8.15	7.521

**Table 3:** Optimised chromatographic conditions, system suitability, and recovery study.

Parameter	Chromatographic conditions		
Instrument	SHIMADZU LC-20AT Prominence liquid chromatograph		
Column	WELCHROM C_18_ column (250 mm × 4.6, 5 *µ*m)		
Detector	SHIMADZU SPD-20A Prominence UV-Vis detector		
Diluents	Mobile phase		
Mobile phase	Water : methanol : glacial acetic acid (65 : 34 : 1 v/v)		
Flow rate	1 mL/min		
Detection wave length	UV at 310 nm		
Run time	15 minutes		
Column back pressure	114 kgf		
Temperature	Ambient temperature (25°C)		
Injection volume	20 *µ*L		
System suitability parameters			
Retention time (min.)	6.617		
Theoretical plates [th.pl] (Efficiency)	7881		
Tailing factor (asymmetry)	1.209		

Recovery study of P-coumaric acid			

Serial number	** 1**	**2**	**3 **

Level of spiking of standard	**50%**	**100%**	**125%**
Amount of reference standard added to preanalysed samples (*µ*g/mL)	2.4	4.8	6.0
Amount found^*^ (*µ*g/mL)	7.19	9.58	10.77
Percentage of recovery^*^ ± S.D (*N* = 3)	99.86	99.80	99.72

^*^Mean of three determinations.

**Table 4:** Linearity analysis of HPLC method.

Serial number	Concentration (*µ*g/mL)	Area of standard P-coumaric acid
**1**	0	0
**2**	2	963
**3**	4	1762
**4**	6	2687
**5**	8	3540
**6**	10	4562

**Table 5:** Determination of concentration of P-coumaric acid acid in methanolic extract of durva grass from its precision study.

Trail number	P-coumaric acid		
	Area	Concentration (*µ*g/mL)	Amount (mg/100 mL extract)
1	2157	4.788	0.478
2	2149	4.77	0.477
3	2159	4.792	0.479
4	2164	4.803	0.480
5	2155	4.783	0.478
6	2174	4.82	0.482

		Mean	0.479
Result		S.D	8.5713
		%RSD	0.3969
